# Modified Silver Impregnation Method for Basal Membranes in Renal Biopsies

**DOI:** 10.7759/cureus.30171

**Published:** 2022-10-11

**Authors:** Hristo Popov, George S Stoyanov, Peter Ghenev

**Affiliations:** 1 General and Clinical Pathology/Forensic Medicine and Deontology, Medical University of Varna, Varna, BGR; 2 General and Clinical Pathology, St. Marina University Hospital, Varna, BGR

**Keywords:** methenamine, staining protocol, silver impregnation, renal biopsy, nephropathology

## Abstract

Silver impregnation methods are essential in biopsy interpretation in nephropathology with regard to visualizing the basal lamina and its associated changes. The most widely used methods, mainly Jones methenamine impregnation, are time-consuming in their protocols and require multiple microscopy control points. In this report, we propose an alternative, modified method for silver impregnation with methenamine solution with a significantly shorter protocol time and good staining quality, allowing for proper interpretation of basal lamina changes in the glomeruli and blood vessels. Furthermore, unlike some other modified techniques, our proposed protocol does not include microwaving of the solutions but rather a thermostat is used, thereby reducing fire hazards. Implementing the protocol in our everyday practice has reduced sample processing time while not negatively impacting biopsy interpretation.

## Introduction

Renal biopsies are a standard pathological method with significant methodological specifics [1]. Interpretation of the basal membranes and their characteristics is critical for distinguishing between several types of glomerulonephritis, such as stage membranoproliferative and membranous nephropathy [1]. A suitable method for the visualization of the basement membranes of the glomeruli in renal biopsies and one of the most widely available methods for silver impregnation is the Jones silver stain, also referred to as methenamine periodic acid-Schiff (PAS) stain [2,3].

In this report, we propose a modified method of silver impregnation, suitable both for glomerular capillary basement membranes and tubular basement membranes.

## Technical report

General safety precautions

Technicians should wear safety gloves, goggles, and laboratory clothing covering all exposed skin areas. Inhalation and direct contact with the reagents should be avoided, especially with sodium thiosulfate, which is toxic if swallowed, and highly irritating to the skin, eyes, and respiratory tract; silver nitrate is highly irritating to the skin, eyes, and the gastrointestinal system, and is also a probable carcinogen.

Do not use metal instruments to avoid silver mirror reaction, depleting the silver in the working solution and decreasing staining reaction quality due to low silver deposition.

Tissue fixation

Fresh tissues should be fixated in a solution prepared on the spot, consisting of 1 gram of picric acid, 150 ml of 80% ethyl alcohol, and 60 ml of 40% formalin. For proper fixation of core needle biopsy specimens, tissues should remain in the fixative for 12-20 hours.

Slide preparation

Embed the fixated tissues in paraffin, cut them into 2 µm-thick sections using a conventional microtome, and fix them on glass slides.

Required reagents for staining are as follows:

1. 5% Silver nitrate solution

2. 2% Borax solution

3. 3% Methenamine solution

4. 1% Periodic acid

5. 0.25% Gold chloride

6. 3% Sodium thiosulfate

Mix 40 ml of the 3% methenamine, 5 ml of the 2% borax solution, and 5 ml of the 5% silver nitrate solution. To avoid a silver mirror reaction, the solution should be prepared by mixing the reagents in the order they are listed. The prepared working solution of silver methenamine is placed in a Coplin jar and placed on a thermostat at 60 °C for 30 minutes.

Staining methodology

1. Deparaffinize and hydrate the slides with distilled water. Slides can be kept in distilled water for prolonged periods

2. Place slides in periodic acid for 15 minutes

3. Wash three times with distilled water

4. Place the slides in the Coplin jar with the preheated working solution of silver methenamine and return them to the thermostat at 60 °C for 90 minutes

5. Remove slides from the thermostat and let them cool for 15 minutes

6. Wash with distilled water

7. Place slides in 0.25% gold chloride solution until the tissue sections turn gray. Control staining after 30 seconds have elapsed under a light microscope; if needed, return them to the gold chloride solution, but for no more than a minute total in the solution

8. Wash three times with distilled water

9. Dip the slides in sodium thiosulfate solution three times

10. Wash the slides under running water for two minutes

11. Bring them to absolute alcohol

12. Dip them in xylene

13. Mount

Expected results and control tissues

**Figure 1:**
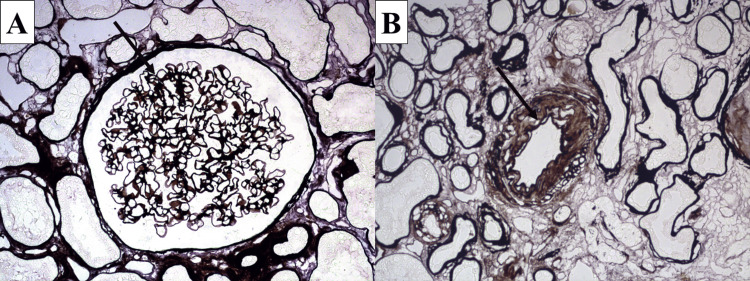
Control staining and expected results A: glomerular capillary basement membranes stained in brownish-black (arrow), modified silver impregnation, original magnification x400; B: elastic membrane of artery stained in black (arrow), modified silver impregnation, original magnification x400

**Figure 2:**
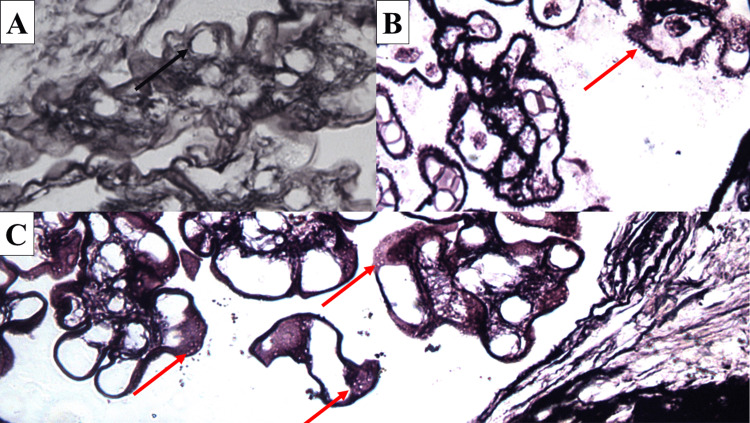
Effectiveness of the staining method in distinguishing glomerular basement membrane changes Tram-track sigh (arrow) in membranoproliferative nephropathy (A), spiking (arrow) and basement membrane vacuoles (arrows) in membranous nephropathy (B and C); modified silver impregnation, original magnifications x1000

**Figure 3:**
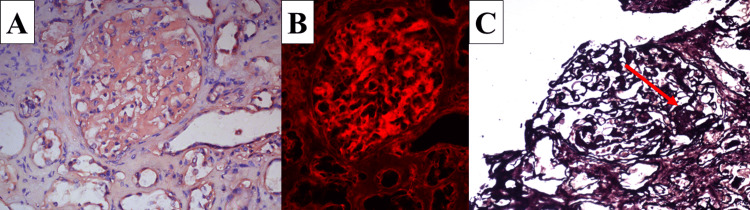
Amyloidosis A: diagnostic Congo red staining; B: diagnostic rhodamine immunofluorescence on Congo red-stained slide; C: nonspecific black stain of the amyloid (arrow) using the modified silver impregnation method; original magnifications x400

**Figure 4:**
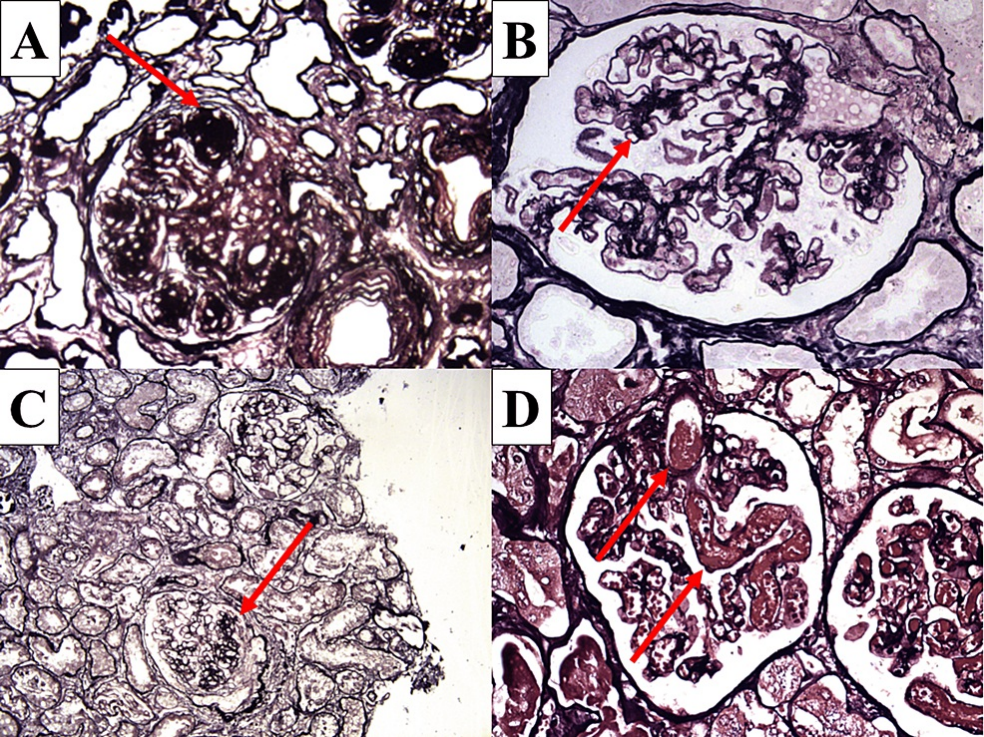
Effectiveness of the staining method in distinguishing mesangial changes and fibrin Nonspecific black staining of proteinaceous deposits (arrow) in monoclonal immunoglobulin deposition (A), diabetic nephropathy class two (B) and focal segmental glomerulosclerosis (C), brick brown staining of fibrin (arrows) in thrombotic microangiopathy (D); original magnifications x200 for A and C, x400 for B and D

Sections from healthy kidneys are optimal for staining control. Basement membranes stain in brownish-black (Figure 1A) and elastic membranes of arterial vessels stain in black (Figure 1B). The method is also specific and sensitive enough to distinguish basement membrane changes in glomerulonephritis showing good differentiation in tram-tracking for membranoproliferative glomerulonephritis (Figure 2A) and perpendicular basement membrane projections (spiking) and vacuoles in membranous nephropathy (Figures 2B, 2C). The staining method is nonspecific for amyloidosis (Figure 3) and, while again nonspecific, underlines the changes well in monoclonal immunoglobulin deposition (Figure 4A), diabetic nephropathy class two (Figure 4B) and focal segmental glomerulosclerosis (Figure 4C), where proteinaceous deposits stain in black. Again, a nonspecific but good distinguishing potential is seen in thrombotic microangiopathy, where fibrin stains in brick brown (Figure 4D).

## Discussion

The proposed methods use a suitable fixator for the tissues, allowing for optimal histochemical staining with other stains, immunohistochemistry, genetic analysis, and electron microscopy. Other fixatives, such as neutral 10% formalin, lead to a marked contraction of dense fabric and low contrast in staining [1]. Our proposed modified silver impregnation is a sensitive method for visualization of tubular basal membranes, glomerular capillary basement membranes, and elastic fibers in the walls of small arteries, as well as subintimal fibrosis. The resulting stained tissue sections are of high quality and have good contrast.

Most readily available silver impregnation methods include a counterstain, which requires more reagents, prolongs the staining time, and allows for other mistakes in these protocols [4,5]. Furthermore, the slides stained in such a manner do not give any additional information about tissue pathology. Other than minimizing the reagents and hence the cost and possibility of errors in the staining protocol, our proposed method is significantly shorter in the time needed to carry out the methodology when compared to the most widely used silver impregnation method in nephropathology - the Jones silver stain [2]. Furthermore, some modifications of the Jones method include microwaving the slides in the solution in a loosely covered Coplin jar, which is a hazard for vapor production and explosion if the jar lid slips and seals under boiling temperatures [1]. The Jones method uses multiple microscopy control points in the protocol, reaching upwards of 10 for a single slide, and may require de-staining with potassium ferricyanide, which is a mild irritant but may produce highly toxic hydrogen cyanide vapors in acidic environments as used in the protocol [2]. In this aspect, our protocol not only involves significantly fewer steps and, therefore, faster, but also has fewer control points, no more than two per slide, which are several seconds from one another and not in between 10 minutes, thereby further reducing technician exposure to potential irritants and toxins. As seen in the provided figures, the reaction is easy to control and provides good staining results, adequate for differentiating basal membrane changes. Furthermore, unlike some modifications of the Jones procedure, our proposed protocol does not include microwaving of the solution and slides, which, depending on microwave type and power, can produce varying results while also being a fire and explosion hazard [4].

## Conclusions

The proposed staining methodology, while not differing significantly from the established silver impregnation methods in nephropathology, has several advantages. The first of these is the significantly shortened staining time, minimal control points for microscopy, and equivalent staining results regarding basal lamina differentiation. In addition, thermostat incubation reduces the risks of fire hazards. The only way in which the visualization differs significantly is the lack of counterstaining, which does not reduce the quality of the interpretation of the reaction. In our practice, implementing the method has shortened the time for biopsy processing without negatively impacting it.
